# Improving current understanding of cognitive impairment in patients with a spinal cord injury: A UK-based clinician survey

**DOI:** 10.1080/10790268.2024.2426313

**Published:** 2024-11-22

**Authors:** Hamish Patel, Daniel Blackburn, Ram Hariharan, Krishnan Padmakumari Sivaraman Nair, Simon M. Bell

**Affiliations:** 1 Department of Clinical Neurology, Royal Hallamshire Hospital, Sheffield, UK; 2 Sheffield Institute for Translational Neuroscience (SITraN), The University of Sheffield, Sheffield, UK; 3 Yorkshire regional spinal injuries centre, Pinderfields Hospital Wakefield, Wakefield, UK; 4 Princess Royal Spinal Injuries and Neurorehabilitation Centre, Northern General Hospital, Sheffield, UK

**Keywords:** Spinal cord injury, Cognitive impairment, SCI and cognition, MOCA, MMSE

## Abstract

**Context:**

Emerging data suggests that patients with a spinal cord injury (SCI) have a higher risk of developing cognitive impairment. The true incidence of cognitive impairment in this group is unclear due to the difficulty in administering commonly used assessment tools, which are dependent on functional abilities e.g. drawing and writing.

**Methods:**

A 16-question online survey, that aims to understand current practices on the assessment of cognition and the limitations of currently available tools, was sent via a Research Network Group to British Association of Spinal Cord Injury Specialists (BASCIS) registered clinicians at each of the 12 Spinal Injuries Centers in the UK and Ireland.

**Results:**

41 responses from 11 different SCI centers, with most from clinicians who have worked with SCI patients for over 10 years. 68% felt that there was a higher incidence of cognitive impairment in those with an SCI. However, 15% reported not using tools to screen for cognitive impairment, primarily due to lack of time, lack of guidelines, and physical disabilities affecting the ability to complete tests. When used, the most commonly utilized tools were those that rely on intact hand function.

**Conclusions:**

Clinicians report a higher incidence of cognitive impairment in those with an SCI. However, currently used tools are not always appropriate, and patients with impaired hand function may be under-represented or undiagnosed. Further work is needed for a more standardized assessment tool to ensure that these patients receive appropriate diagnosis and management, particularly since cognitive impairment in this group can increase care needs and reduce engagement with rehabilitation.

## Introduction

Spinal cord injury (SCI) is a devastating neurological condition with a rising prevalence in the elderly population (1, 2). Emerging data suggests that people with an SCI are at a higher risk of developing cognitive impairment, and this risk may be up 13 times higher than in those without an SCI (3). These impairments particularly affect attention and executive functioning (4). Furthermore, people with an SCI are at a higher risk of developing Alzheimer’s disease, approximately doubling the risk of early-onset Alzheimer’s disease in those aged 45–64 years old (5).

Traumatic brain injury (TBI) occurring at the time of the SCI is often thought to contribute to the development of cognitive impairment (6). However, not all studies demonstrate a clear relationship (3, 7). Other mechanisms have been proposed including neuroinflammation, chronic hypotension, polypharmacy, substance abuse, and the development of psychiatric disorders, amongst others (see Alcántar-Garibay *et al*. 2022 for an extensive review) (8).

When cognitive impairment is suspected, various psychometric tests can be used as a screening tool to support a diagnosis. The most commonly used tools include the Montreal Cognitive Assessment (MoCA), the Mini-Mental State Examination (MMSE) and the Clock Drawing Test (CDT) (9). These tests require the patient to perform various tasks, such as drawing a clock face. However, the ability to complete these tests may be impaired in people with an SCI, particularly a higher cord injury where hand function is impaired. This could lead to a delay in the diagnosis of early cognitive impairment in this group. This is likely to be exacerbated by the changing demographics of people with SCI, where there is now a higher prevalence amongst elderly people (1), who are already at a higher risk of developing cognitive impairment given their age. There is an increasing importance in the early diagnosis of neurodegenerative conditions, such as Alzheimer’s disease, given the progress in disease-modifying therapies that are likely to have a greater impact earlier in the disease process (6).

Furthermore, there is no consensus on the most appropriate tools to use in this group, and there remains a significant heterogeneity in the literature, with nearly all studies utilizing a different combination of tools (10).

The combination of lack of guidelines, and the difficulty in administering common cognitive assessment tools, may mean that this problem goes unrecognized in a patient group that is already at a higher risk of developing cognitive impairment. Cognitive impairment in this group has shown to result in poorer motor outcomes and longer acute rehabilitation needs, emphasizing the importance of early recognition and diagnosis (11).

Given the lack of consensus on the most appropriate screening tests for people with SCI, or lack of guidance on when to perform the assessment, we developed a questionnaire to better understand the experience of SCI specialists with regards to screening for cognitive impairment.

## Methods

Ethical approval was gained through the University of Sheffield Ethics committee, reference 046972, and approved on the 7th June 2022.

An electronic 16-question survey was sent to all 12 specialist SCI centers within UK and Ireland via a Research Network Group. Informed consent was gained through the questionnaire link. The submissions were pseudo-anonymized. The questionnaire went live on 9th June 2023, receiving 23 responses. Initial results were presented at the 2023 *British Society of Physical and Rehabilitation Medicine* (BSPRM) and *British Association of Spinal Cord Injury Specialists* (BASCIS) Joint Annual Conference (BSPRM-BASCIS) (12) which led to a further 18 responses. Responses were collected until October 2023. Appendix 1 includes the full list of questions including responses available and response rate.

## Results

The survey received 41 responses from 11 different SCI centers in the UK and Ireland, one response from the Netherlands and one from Australia (Table 1). Most responses were from consultant physicians (11, 27%), followed by allied healthcare professionals (AHP) (11, 27%), and clinical psychologists (6, 15%). Responses were also received from junior doctors, nurses, and case managers. Most responses were from people who had worked with SCI patients for >10 years (58.6%). They saw an average of 153 patients per year (95% CI 153.4 ± 52.9) (Table 2).

**Table 1:** Responses from SCI centers. Three responses from ‘Other’ were not specified.

Spinal cord injury center	Number of responses
National Spinal Injuries Centre	14
The London Spinal Cord Injury Centre	4
Welsh Spinal Cord Injury Rehabilitation Centre	4
National Spinal Injuries Unit (Dublin)	3
Other (not specified)	3
The Yorkshire Regional Spinal Injuries Centre	2
The Queen Elizabeth National Spinal Injuries Centre	2
Princess Royal Spinal Injuries Centre	2
NorthWest Regional Spinal Injuries Centre	1
The Golden Jubilee North East Regional Spinal Injuries Centre	1
Midlands Centre for Spinal Injuries	1
Duke of Cornwall Spinal Treatment Centre	1
Salford Royal (Acute SCI services)	1
The Rehabilitation Centre De Hoogstraat (Netherlands)	1
Royal Rehab Spinal Cord Injury Unit (Sydney)	1

**Table 2:** Demographics of respondents, including profession, average number of years worked with people with SCI, and average number of patients seen per year.

Profession	Average years worked and range	Average number of patients seen and range	Do you believe there is a higher incidence of cognitive impairment in people with an SCI? (% of yes and no)	Would you find it useful to have a standardized assessment tool?
Consultant (11)	21 (4–20+)	178 (4–500)	72% Yes	100% Yes
			28% No	0% No
Allied Healthcare professionals (11)	11 (0–20+)	115 (25–300)	64% Yes	90% Yes
			36% No	10% No
Clinical psychologist (6)	10 (0–20)	63 (50–100)	100% Yes	83.3% Yes
			0% No	16.6% No
Nurse (4)	11 (0–20)	133 (50–300)	75% Yes	100% Yes
			25% No	0% No
Specialist nurse (3)	17 (11–20+)	500 (300–800)	66% Yes	100% Yes
			33% No	0% No
Junior doctor (3)	4.5 (4-5)	45 (30–50)	66% Yes	100% Yes
			33% No	0% No
Case manager (2)	12 (4-20+)	100 (100)	100% No	100% Yes
			0% Yes	0% No
Medical secretary (1)	20 (20)	n/a	100% No	0% Yes
			0% Yes	100% No

Percentage of respondents who believe that there is a higher incidence of cognitive impairment in people with SCI compared to those without.

Percentage of respondents who would find it useful to have a standardized assessment tool to monitor the development of cognitive impairment in people with an SCI.

**Figure 1:**
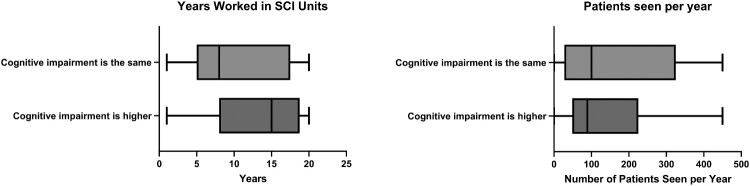
Respondents who felt cognitive impairment was higher in those with SCI, relative to their experience (number of years worked and number of patients seen).

We asked if respondents felt that there was a higher incidence of cognitive impairment in those with an SCI compared to those without, with 68% responding Yes. Respondents who felt the incidence of cognitive impairment was higher in SCI patients, on average, had worked for more years in SCI units (13.2 vs 9.8 years) and saw fewer patients (135 vs 155 per year) (Fig. 1). When responses were separated based on profession, consultant physicians, nurses, and clinical psychologists thought that the incidence of cognitive impairment was higher than AHP, specialist nurses, and junior doctors.

Of those that felt there was a higher incidence of cognitive impairment in those with an SCI, the domains they felt that were most affected were memory (90%), executive function (69%), verbal fluency (24%), visuospatial awareness (14%), language (4%), and speed of processing (4%). When asked how cognitive impairment affected the needs of SCI patients, 88% thought that care needs were increased, 84% felt pressure sores and infections were more likely, 52% felt it affected mobility, and 17% felt it impacted on engagement with rehabilitation.

When asked about the availability of neuropsychological assessment for patients with an SCI, 39% reported that their service does not have a dedicated neuropsychology service, and interestingly, 15% responded that they do not use any neuropsychological tools to screen for cognitive impairment in this group. Reasons included a lack of time in busy clinics, a lack of guidelines, and physical disabilities affecting the ability to complete the test.

In services where neuropsychological tools are used, the most commonly used tools were the Montreal Cognitive Assessment (MoCA, 77%), Addenbrooke’s Cognitive Examination (ACE, 37%), and Mini-Mental State Examination (MMSE, 34%). Table 3 displays all tools reported to be used by respondents.

**Table 3:** Currently used neuropsychological tools to screen for cognitive impairment in people with an SCI.

Neuropsychogical tool used	Number of respondents
Montreal Cognitive Assessment (MoCA)	29 (70%)
Addenbrooke’s Cognitive Exam (ACE)	13 (32%)
Mini-Mental State Examination (MMSE)	12 (29%)
Blind MoCA	4 (10%)
Abbreviated Mental Test (AMT)	4 (10%)
Edinburgh Cognitive and Behavioural ALS Screen (ECAS)	2 (5%)
Repeatable Battery for the Assessment of Neuropsychological Status (RBANS)	2 (5%)
Oxford cognitive screen (OCS)	1 (2%)
Neuropsychiatry Unit Cognitive Assessment Tool (NUCOG)	1 (2%)
Wechsler Adult Intelligence Scale Fourth Edition (WAIS-IV)	1 (2%)
Rivermead Behavioural Memory Test (RBMT)	1 (2%)
Behavioural Assessment of the Dysexecutive Syndrome (BADS)	1 (2%)

In centers where neuropsychological tools are not used, people with suspected cognitive impairments are referred to internal memory services (within their SCI service) (50%), external memory services (12.5%), or back to their GP for referral to an appropriate service (12.5%). The remaining respondents would arrange further investigations, such as brain imaging, prior to onward referral. The waiting time to be reviewed in a memory service ranged from 1 to 18 months, but only 7 respondents answered this question.

Overall, 93% of respondents felt that it would be useful to have a standardized assessment tool to screen for cognitive impairment in those with an SCI. Health care professionals who felt that standardized assessment was not needed included a clinical neuropsychologist, AHP, and medical secretary.

## Discussion

The incidence of cognitive impairment following an SCI has been reported to be between 10% and 60% (13). In our survey, 68% felt that there is a higher incidence of cognitive impairment in this group, compared to the general population. However, we found that many centers do not routinely screen for cognitive impairment, and therefore the true incidence is not known.

In services where neuropsychological tools are used, the most commonly used tools are the MoCA, ACE, and MMSE. However, all three of these tools require intact hand function to adequately complete. Drawing or writing tasks contribute to a large proportion of the overall score in these tasks at 20% for the MoCA, 17% for the ACE, and 20% for the MMSE. In cases where hand function is impaired, tests can be modified and adapted (14). In practice, clinicians may not be able to confidently interpret the results of modified tests, or opt not to screen patients that are unable to complete the test. Furthermore, test modifications often need to be made ‘in the moment’, adapting the test to the individual needs of the patient (15) which may affect the validity of the test, and this is reflected in the literature, highlighting a difficulty in developing validity data for these adapted tests (15). Tests that have been adapted may miss early or subtle changes of cognitive impairment. For example, the MoCA has been adapted for use with individuals with visual impairments (MoCA-Blind or MoCA-22), removing four vision-dependent items, 3 of which rely on intact hand function. Analysis shows that the MoCA-Blind has a lower sensitivity for detecting Alzheimer’s disease and an even lower sensitivity for detecting mild cognitive impairment (16).

Furthermore, test components which rely on intact hand function, such as the clock drawing test, are primarily assessing visuospatial and executive function (17). In particular, these tests are assessing higher level executive function such as set shifting and multi-tasking, which can be impaired even in the earliest stages of mild cognitive impairment (18).

Given that visuospatial and executive function are typically impaired early in dementia (19, 20), cognitive impairment or dementia would likely be diagnosed later in this group. This will clearly have consequences for rehabilitation after an SCI. With recent developments in disease-modifying therapies for Alzheimer’s disease, inadequate screening may lead to delay in people with SCI receiving treatments. People living with SCI are also at an increased risk of cardiovascular disease (21), mood changes (22) and social isolation (23). These are all modifiable risk factors for developing dementia (24) and early identification of cognitive impairment may affect the level of aggressiveness with which the SCI physician may want to address these issues. There is also a belief amongst our survey responders that cognitive impairment increases the risk of both infections and pressure ulcers. Accurate and timely identification of cognitive impairment may mean that patient care plans can be adapted for higher vigilance of these complications. However, it is important to consider that complications of an SCI, such as infections and pressure ulcers, may lead to fluctuating cognitive impairments, such as delirium. Although this can bias the findings of any cognitive tests that are performed, experienced clinicians would usually consider and recognize this during the assessment of cognitive impairment.

Our survey showed that not all centers have a dedicated neuropsychology service, and in these centers, people with suspected cognitive impairment may be referred to memory services. Nationally, the average wait time for a memory clinic referral is 21.6 weeks (ranging from 0 to 117 weeks) (25). Furthermore, memory clinics are typically within acute trusts that may not be easily accessible for people with an SCI. In our survey, the wait time ranged from 1 to 18 months, although this data is based on a small number of responses. Rehabilitation following an SCI is complex and intensive. The process of learning new skills, adapting to new limitations, and re-integrating into society may be hindered by cognitive impairments (26). Prolonged waiting times for a cognitive assessment may be further detrimental to this process. Indeed, our survey respondents felt that cognitive impairments can increase care needs and reduce engagement with rehabilitation. Furthermore, impaired cognitive function during acute rehabilitation is associated with poorer psychological well-being and social participation following discharge (27, 28).

A major barrier to screening for cognitive impairment in this group is the lack of a standardized tool that is appropriate for all patients with SCI. This is clearly an unmet need, with 93% reporting in our survey that they would find it useful to have a standardized assessment tool. One approach has been to use tools which do not rely on intact hand function, such as the Edinburgh Cognitive and Behavioural ALS Screen (ECAS). However, only one study has looked at its use in patients with SCI, and further validity studies are required (29). Alternatively, there may be a role for emerging technology, such as the use of a brain-computer interface (30). For example, a system which uses Automatic Speech Recognition to extract linguistic measures seen in the speech and language of patients with cognitive impairment has previously been trialed in people with a stroke (31, 32).

Nevertheless, at present, there are no neuropsychological tools designed specifically for people with SCI, and current strategies rely on adapting currently available tools, with limited evidence on validity. Therefore, a more unified approach is needed, particularly given the significant heterogeneity in what is currently being used in clinical research and practice.

Our survey is limited by a modest number of responses but has good representation from the SCI centers within the UK. The survey did not ask respondents to specify their exact job role. For example, AHP consists of fourteen different professions, and separating these with responses may be useful to determine if certain health professionals have more or less experience in identifying cognitive impairments in this group. Furthermore, in an endeavor to make the questionnaire as inclusive as possible, the survey was open to various members of the multi-disciplinary team, and the responses from non-clinical members may be different to that of clinicians. Lastly, a limitation of questionnaire-based studies is a limited response rate. Our survey received a good response rate to each question (see Appendix 1), taking into account qualifying questions.

## Conclusion

Patients with SCI may be at a higher risk of developing cognitive impairment. However, cognitive impairment is not routinely screened for in this patient group. Furthermore, many of the commonly available tools may not be appropriate for all patients with an SCI, particularly those with impaired upper limb function, likely leading to later diagnosis and reduced memory impairment identification. This can be detrimental to rehabilitation and re-engagement with society. There is a clear desire in the UK for a standardized assessment tool, and the developments in brain-computer interfaces may be a solution to this.

## Appendix

Survey questions, available responses, and number of responses out of 41 total respondents

**What is your role in the spinal injuries unit you work in? (41 responses)**

*Multiple choice, single select*

- Consultant
- Specialist nurse
- Junior doctor
- Psychologist
- Allied health professional
- Other (free text)

**How many years have you worked with people with spinal injuries? (41 responses)**

*Multiple choice, single select*

- 0–1 Years
- 4–5 Years
- 6–10 years
- 11–20 years
- Over 20 years

**How many patients with spinal injuries would you see in a year? (40 responses)**

- Free text

**Which spinal injuries unit do you work in? (41 responses)**

- Free text

**In your experience is there a higher incidence of cognitive impairment or dementia in people living with a spinal cord injury, when compared to the general population? (41 responses)**

- Yes
- No

**If you answered Yes to the above, what domain do you believe is most commonly affected? (29 responses)**

*Multiple choice, multi-select*

- Memory
- Executive function
- Visuospatial awareness
- Language
- Verbal fluency
- Other (free text)

**In your experience how does the cognitive impairment affect the life of people living with Spinal cord injury? (39 responses)**

*Multiple choice, multi-select*

- No impact
- Increase in care needs
- Increase in complications like pressure ulcers and infections
- Reduction in mobility
- Other (free text)

**Does your spinal cord injury service have a dedicated neuropsychology service? (41 responses)**

- Yes
- No

**If you answered No to question 8, is there a specialist service to which you can refer to e.g. local memory service. If you answer yes to this question, please elaborate on the service that is offered. (12 responses)**

- Free text

**How long does it take for a patient to be seen by neuropsychology/local memory service once referred (please answer in months)? (36 responses)**

- Free text

**Do you know of any neuropsychological assessment tools that can be used to assess for cognitive impairment or dementia in patients with a spinal cord injury? (40 responses)**

- Free text

**Does your service currently use any of these neuropsychological assessment tools to assess for cognitive impairment or dementia in patients with a spinal cord injury? (40 responses)**

- Yes
- No

**If you answered Yes to the above, does your service currently use any of below cognitive assessment tools? (35 responses)**

*Multiple choice, multi-select*

- Montreal Cognitive Assessment tool (MOCA)
- Blind-MOCA
- The Mini Mental Status Examination (MMSE)
- Addenbrooke's Cognitive Examination (ACE)
- Abbreviated Mental Test (AMT)
- Edinburgh Cognitive and Behavioral ALS Screen (ECAS)
- Other (free text)

**If you answered No to the above, could you explain why? (6 responses)**

*Multiple choice, multi-select*

- Physical disabilities affecting the ability to do the test e.g. drawing and writing
- Lack of time
- Not in the current practice guidelines
- Lack of engagement by the patients
- Other (free text)

**If you identify a patient with possible cognitive impairment or dementia in a patient with a spinal cord injury, what do you do? (41 responses)**

*Multiple choice, single select*

- Arrange for further tests such as brain imaging
- Refer to a specialist internal memory service
- Refer to a specialist external memory service
- Ask GP to refer to appropriate service
- Other (free text)

**Would you find it useful to have a standardized assessment tool for monitoring the development of cognitive impairment or dementia in patients with a spinal cord injury? (41 responses)**

- Yes
- No
